# Altered Expression of *MGMT* in High-Grade Gliomas Results from the Combined Effect of Epigenetic and Genetic Aberrations

**DOI:** 10.1371/journal.pone.0058206

**Published:** 2013-03-11

**Authors:** João Ramalho-Carvalho, Malini Pires, Susana Lisboa, Inês Graça, Patrícia Rocha, João Diogo Barros-Silva, Joana Savva-Bordalo, Joaquina Maurício, Mário Resende, Manuel R. Teixeira, Mrinalini Honavar, Rui Henrique, Carmen Jerónimo

**Affiliations:** 1 Cancer Epigenetics Group, Research Center of the Portuguese Oncology Institute-Porto, Porto, Portugal; 2 Cancer Genetics Group, Research Center of the Portuguese Oncology Institute-Porto, Porto, Portugal; 3 Department of Genetics, Portuguese Oncology Institute-Porto, Porto, Portugal; 4 Department of Medical Oncology, Portuguese Oncology Institute-Porto, Porto, Portugal; 5 Department of Pathology, Portuguese Oncology Institute-Porto, Porto, Portugal; 6 Department of Pathology and Molecular Immunology, Institute of Biomedical Sciences Abel Salazar (ICBAS), University of Porto, Porto, Portugal; 7 Department of Neurosurgery, Local Health Unit of Matosinhos - Hospital Pedro Hispano, Matosinhos, Portugal; 8 Department of Pathology, Local Health Unit of Matosinhos - Hospital Pedro Hispano, Matosinhos, Portugal; University of Navarra, Spain

## Abstract

*MGMT* downregulation in high-grade gliomas (HGG) has been mostly attributed to aberrant promoter methylation and is associated with increased sensitivity to alkylating agent-based chemotherapy. However, HGG harboring 10q deletions also benefit from treatment with alkylating agents. Because the *MGMT* gene is mapped at 10q26, we hypothesized that both epigenetic and genetic alterations might affect its expression and predict response to chemotherapy. To test this hypothesis, promoter methylation and mRNA levels of MGMT were determined by quantitative methylation-specific PCR (qMSP) or methylation-specific multiplex ligation dependent probe amplification (MS-MLPA) and quantitative RT-PCR, respectively, in a retrospective series of 61 HGG. *MGMT*/chromosome 10 copy number variations were determined by FISH or MS-MLPA analysis. Molecular findings were correlated with clinical parameters to assess their predictive value. Overall, *MGMT* methylation ratios assessed by qMSP and MS-MLPA were inversely correlated with mRNA expression levels (best coefficient value obtained with MS-MLPA). By FISH analysis in 68.3% of the cases there was loss of 10q26.1 and in 15% of the cases polysomy was demonstrated; the latter displayed the highest levels of transcript. When genetic and epigenetic data were combined, cases with *MGMT* promoter methylation and *MGMT* loss depicted the lowest transcript levels, although an impact in response to alkylating agent chemotherapy was not apparent. Cooperation between epigenetic (promoter methylation) and genetic (monosomy, locus deletion) changes affecting *MGMT* in HGG is required for effective *MGMT* silencing. Hence, evaluation of copy number alterations might add relevant prognostic and predictive information concerning response to alkylating agent-based chemotherapy.

## Introduction

O^6^-methylguanine-DNA methyltransferase (MGMT) is a DNA repair enzyme that catalyzes the transfer of mutagenic and cytotoxic adducts from O^6^-guanine in DNA [1]. Following the incorporation of the alkyl group by MGMT, the enzyme is irreversibly inactivated and targeted for degradation, thus requiring *de novo* protein synthesis to sustain the enzyme activity. If left unrepaired, O^6^-guanine preferentially couples with thymidine during DNA replication, thus triggering the mismatch repair (MMR) mechanisms [2]. Hence, through removal of alkyl groups from guanines, MGMT safeguards the cells against mutagenesis and malignant transformation [3]. Indeed, *MGMT* deregulation may play an important role in carcinogenesis because tumors frequently display lower expression levels than their tissues of origin [2]. However, *MGMT* downregulation is not only associated with increased risk of tumorigenesis but also with improved sensitivity to alkylating chemotherapeutics [3].

In tumors, *MGMT* downregulation appears to be mostly due to aberrant promoter methylation [2]. Indeed, the *MGMT* gene has a large CpG island, comprising more than 90 CpG sites, encompassing a minimal promoter and an enhancer region [4]. The region spanning from −552 to +289 from the transcription start site is critical for DNA methylation-associated silencing [5], [6] and has been correlated with lower or absent mRNA expression, lower/absent MGMT protein levels [7], [8] and diminished/loss of enzyme activity [7]. Importantly, *in vitro* treatment of malignant cell lines with demethylating agents restores *MGMT* expression [9].

A wide spectrum of human tumors displays *MGMT* hypermethylation, including gliomas [6], lymphomas [10], colon cancer [11], head and neck cancer [12], testicular cancer [13], and retinoblastoma [14]. Glioblastoma multiforme (GBM) is the most common primary brain tumor and it is associated with high morbidity and mortality [15]. GBM is notorious for its resistance to therapy and its ability to infiltrate into adjacent normal brain tissue, rendering the condition incurable by surgery [16]. In spite of progress in treatment, median survival for GBM patients is only 12 to 15 months [16]. *MGMT* promoter methylation acts as a chemosensitizer in GBM, by reducing its expression, consequently enhancing the cytotoxic effects of alkylating drugs and predicting a favorable outcome in patients who are exposed to alkylating agent chemotherapy in combination with radiotherapy [4], [17]. However, it remains unclear how many and which CpGs within the *MGMT* CpG island play a key role on *MGMT* downregulation. More importantly, there is still a need to clearly identify which of those should be analyzed for clinical purposes [18], [19].

Genome-wide comparative genomic hybridization (CGH) analysis of GBM revealed numerous recurrent copy number alterations (CNAs), including loss/deletion of chromosome 10q, to which MGMT is mapped [20], [21]. Loss of heterozygosity at 10q is the most frequent genetic alteration found both in primary and secondary GBM (60–90% of cases) and is seldom found in other gliomas [20]. Interestingly, 10q loss has been associated with shorter survival [22], and patients with high-grade gliomas harboring 10q deletions (detected by CGH) benefited from treatment with Temozolomide, an alkylating agent [23]. Thus, *MGMT* downregulation, irrespective of its molecular basis, is likely to account for improved therapeutic response. In this vein, chromosome 10 monosomy and *MGMT* locus deletion might also be predictors of response to chemotherapy. However, to the best of our knowledge, no comprehensive characterization has been reported of the combined effect of epigenetic and genetic alterations on *MGMT* expression or its eventual clinical impact in high-grade gliomas.

We sought to determine the frequency of aberrant promoter methylation and of chromosome 10 deletion and copy number alterations affecting the *MGMT* locus and to examine how those genomic alterations correlated with transcript levels in high-grade gliomas. In addition, we compared the performance of quantitative methylation-specific PCR (qMSP) and methylation-specific multiplex ligation dependent probe amplification (MS-MLPA) in assessing *MGMT* hypermethylation, and compared the effectiveness of MS-MLPA and fluorescent in situ hybridization (FISH) to detect copy number alterations at the *MGMT* locus. Finally, the putative clinical relevance of those alterations was assessed.

## Methods

### Ethics Statement

This study was approved by the institutional review board of the Portuguese Oncology Institute, Porto (CES-IPOFG-EPE 019/08) and of Hospital Pedro Hispano, Matosinhos (ULS-Matosinhos 101/CE/FC-2009). Because this was a retrospective study, based on archive paraffin-embedded tissue and most patients were deceased at the time when the study was started, no informed consent was procured.

### Patients and Samples

High-grade gliomas [anaplastic astrocytoma (AA) and GBM], diagnosed from 2000 to 2011, not previously treated with chemotherapy and/or radiotherapy and submitted to surgical biopsy or tumor resection were retrieved from the archives of the Departments of Pathology of the Portuguese Oncology Institute, Porto and of Hospital Pedro Hispano, Matosinhos. Only cases with slides and paraffin blocks available for review and sufficient viable tumor for molecular testing were further selected. Relevant clinical data was collected from the patient’s charts.

### Histopathological Analysis

Tissue samples had been collected from resective surgery or biopsy procedures and fixed in 4% buffered formalin and processed for paraffin embedding. Four µm thick sections were obtained for routine stains (hematoxylin and eosin), as well as for immunohistochemistry [human GFAP (clone 6F2, Dako, Glostrup, Denmark), MAP2 (clone HM-2, Sigma, Saint Louis, Missouri, USA), and Ki67 (clone MIB-1, Dako, Glostrup, Denmark)], which were performed according to standard procedures. All cases were reviewed by a neuropathologist (author MH) and classified according to the WHO classification of central nervous system tumors [24].

### Nucleic Acid Extraction

DNA and total RNA were extracted and purified from formalin-fixed, paraffin-embedded tissue samples, using commercially available kits. Briefly, in each case, tumor areas were macrodissected from the five-micrometer thick tissue sections to maximize the proportion of malignant cells (>70%), and subsequently deparaffinized and rehydrated using xylene (DNA) or d-limonene (RNA) and 100% ethanol. The pellet was then resuspended in appropriate buffer, proteinase K added and the solution incubated overnight at 55°C. DNA purification was carried out using the QIAamp® DNA FFPE Tissue Kit (QIAGEN,Germany) according to the manufacturer’s instructions, and stored at −20°C. Total RNA was isolated using Absolutely RNA FFPE Kit (Agilent Technologies, La Jolla, CA) according to the manufacturer’s instructions, and samples were stored at −80°C.

DNA and RNA concentrations and quality were analyzed in a NanoDrop ND-1000 spectrophotometer (NanoDrop Technologies, USA).

### *MGMT* mRNA Quantification Using qRT-PCR

Total isolated RNA was reverse-transcribed using AffinityScript Multiple Temperature cDNA Synthesis Kit (Agilent Technologies, La Jolla, CA). Real-time PCR of *MGMT* transcript was performed on an ABI PRISM 7000 detection system using a Taqman probe for *MGMT* (Hs00172470_m1, Applied Biosystems) and Taqman reagents under default conditions: 95°C for 10 minutes, 40 cycles at 95°C for 15 seconds, and 60°C for 1 minute. Human betaglucuronidase (*βGUS*) was used as endogenous control. All assays were performed in triplicate. Each plate included multiple non template controls and serial dilutions of a positive control (Stratagene QPCR Reference Total RNA, Agilent Technologies, La Jolla, CA) for constructing the standard curve of each plate. To determine the relative expression levels of *MGMT* mRNA in each sample, the values of the target gene were normalized with the values of the internal reference gene to obtain a ratio that was then multiplied by 1000 for easier tabulation (*MGMT*/*βGUS* × 1000).

### *MGMT* Promoter Methylation and Copy Number Analysis by MS-MLPA

MS-MLPA is a semi-quantitative method for methylation profiling using a methylation-sensitive restriction enzyme (*HhaI*) [25]. In addition, this method also provides copy number quantification [26]. The SALSA MS-MLPA ME002-B1 Tumour Suppressor 2 kit (MRC-Holland, Amsterdam, The Netherlands) was used to determine the promoter methylation status and copy number changes of the *MGMT* gene. The kit contains two different probes (MS-MLPA A and MS-MLPA B, which target sequences located at 369 and 119 base pairs from the transcription start site, respectively) that specifically target CpG dinucleotides within the *MGMT* promoter region (Figure S1).

The MS-MLPA assay was performed with 100 ng DNA according to the manufacturer’s protocol. The amplified PCR products were separated by electrophoresis on an ABI PRISM 310 genetic analyser (Applied Biosystems, Foster City, CA, USA), and analyzed using GeneMapper analysis software (Applied Biosystems). Quantification of methylation status was obtained comparing *MGMT* probes relative peak area ratio from the digested sample with those obtained from the undigested sample. Relative copy number information resulted from comparing *MGMT* probes relative peak area ratio with the same ratio obtained from a control sample. For all analyses, the MGMTav score was used either as a binary variable (using the manufacturer cutoff MGMTav ≥0.25, considered to be indicative of methylation) or as a continuous variable, as appropriate [27]. Copy number alterations were classified as loss (0–0.79), normal (0.80–1.19) or gain (>1.2) [25].

### Bisulphite Modification and *MGMT* Promoter Methylation Analysis by qMSP

DNA was modified with sodium bisulphite, using the EZ DNA Methylation-Gold™ Kit (Zymo Research, Orange, CA, USA) according to the manufacturer’s instructions, and used as template for qMSP with a dual-labeled probe complementary to target sequence, as previously described [28]. Two qMSP assays covering two different regions on the CpG island of *MGMT* promoter were performed (Figure S1). The region covered by qMSP1 is located 456 bp upstream of *MGMT* transcription start site (TSS) and the qMPS2 assay targets a region which is downstream of the TSS (51 bp). These two regions are commonly targeted and analyzed by different methods [18], [19]. The primers and probe sequences are listed in Table S1 [8], [29]. Fluorescence-based real-time PCR assays were performed in 96-well plates using an ABI 7000 Sequence Detection System (Applied Biosystems) and carried out in a reaction volume of 20 µL, consisting of 16.6 mM ammonium sulfate; 67 mM trizma preset; 6.7 mM magnesium chloride; 10 mM mercaptoethanol; 0.1% DMSO; 200 µM each of dATP, dCTP, dGTP and dTTP; 600 nM of each primer; 0.4 µL of Rox dye; 200 nM of probe; 1 unit of Platinum Taq polymerase (Invitrogen, Carlsbad, CA), and 2 µl of bisulfite-modified DNA. Each 96-well PCR plate had multiple water blanks, as negative control, and fully methylated DNA, as positive control. All samples were tested in triplicate. The amplifications were performed at 95°C for 2 minutes, followed by 50 cycles of 95°C for 15 seconds and 60°C for 1 minute. To normalize for DNA input in each sample, β*-actin* (*ACTB*) was used as an internal reference gene (primers and probe were designed to amplify a CpG nucleotide-free region. A calibration curve was constructed using serial dilutions of the fully methylated DNA, in order to determine the relative levels of methylated alleles in each sample. The values obtained for the target gene were divided by the value of the internal reference gene. The ratio generated, which constitutes an index of the percentage of input copies of DNA that are fully methylated at the primer-and probe-binding sites, was then multiplied by 1000 for easier tabulation [methylation level = (*MGMT*/*ACTB*) × 1000]. The cutoff value for discrimination between methylation levels was the median ratio for each primer/probe set (methylation positive if higher than the median; methylation negative if equal or lower than the median).

### Fluorescent in situ Hybridization (FISH) Assay

Owing to the unavailability of commercial probes for *MGMT* testing, Bacterial Artificial Chromosome (BAC) clones targeting the *MGMT* gene (RP11 - 1063D3, RP11 - 21C15) were selected using the UCSC Human Genome Browser and obtained from the BACPAC Resources Center [Oakland, USA]. The E. coli bacteria were first grown in LB agarose medium, supplemented with cloramphenicol 12.5 mg/mL, at 37°C, overnight. Then, an individual colony was inoculated in 10 mL of liquid LB medium and incubated at 37°C for 16 hours with continuous agitation. The culture was centrifuged and the pellet used for plasmid DNA extraction using the NucleoSpin® Plasmid kit [Macherey-Nagel, Germany], according to the manufacturer’s instructions. DNA concentration was determined in a NanoDrop ND-1000 spectrophotometer.

After adjusting the plasmid DNA concentration to 10 ng/µL, amplification was carried out using the Illustra GenomiPhi V2 DNA Amplification Kit [GE Healthcare, US], according to the manufacturer’s instructions. Probes were labeled using a nick translation DNA labeling system (Enzo Life Sciences, Exeter, UK). DNA was eluted in 10 µL of Vysis LSI/WCP Hybridization Buffer [Abbott Molecular, Illinois, USA].

FISH analysis for *MGMT* was performed in 4 µm thick tissue sections obtained from representative paraffin blocks of each sample and placed in SuperFrost Plus Adhesion slides (Menzel-Glaser, Braunschweig, Germany). Sample processing, hybridization, and analysis were performed as previously described [30]. The *MGMT* probe was combined with the Vysis centromeric probe for chromosome 10 (CEP10), labeled with SpectrumOrange (Abbott Molecular, Illinois, USA) and applied to each sample. An abnormal signal pattern was considered representative when present in a minimum of 100 morphologically intact, non overlapping nuclei. Only cases with copy number alterations present in 20% or more of the analyzed nuclei were considered positive.

### Statistical Analysis

Methylation and expression levels were compared using Spearman's rank coefficient correlation test. To compare the ranks of methylation in a single sample according to different assay we applied Wilcoxon signed-rank test. Frequencies of expression and of methylation within sample groups were compared using the Chi-square test. The median of *MGMT* methylation and mRNA expression levels in all samples was used as the cut-off value for definition of the *MGMT* methylation status or to categorize into high and low mRNA expression groups, respectively. *MGMT* methylation, expression and copy number data, as well as clinical parameters, were compared within groups using Chi-square or Mann-Whitney tests, as appropriate. Survival analysis was performed using the Kaplan-Meier method and survival curves were compared with the two-sided log-rank test. The Cox model was fitted to assess the prognostic value of the clinical parameters, *MGMT* methylation status, *MGMT* mRNA expression levels, and *MGMT* copy number changes. First, the contribution of each variable was tested univariately. Forward and backward step-wise proportional hazards modeling was then performed to assess the relative and independent prognostic power of each parameter. A p-value <0.05 was considered statistically significant. All analyses were performed using SPSS v15 (IBM Company, Chicago, Illinois, USA).

## Results

### Clinical and Pathologic Data

A total of 61 patients with a median age of 58 years (range: 15 to 80 years) were included in this study. Table S2 provides detailed clinical and pathological data. Only 4 patients underwent stereotactic biopsy procedures, whereas craniotomy resection was performed in the remainder. Histopathological evaluation revealed GBM in 56 patients and AA in five patients. The Karnofsky performance status ranged between 20 and 100. Treatment regimens included radiotherapy or chemotherapy with alkylating drugs (mainly Temozolomide), or a combination of both.

### *MGMT* mRNA Expression Quantification

**Figure 1:**
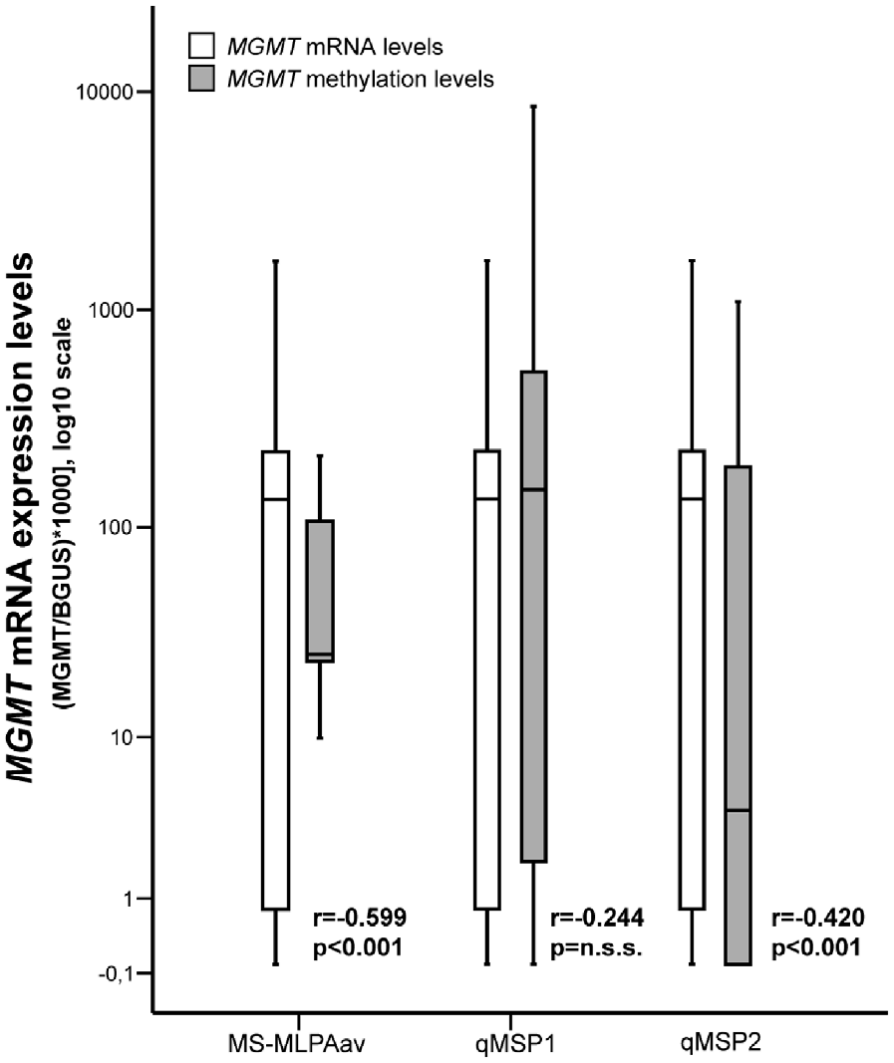
Distribution of *MGMT* promoter methylation and mRNA expression levels according to MS-MLPA average or qMSP techniques in tissue samples of high-grade gliomas (r and p values are provided for each methylation assay).

Firstly, we assessed *MGMT* gene expression levels using quantitative RT-PCR (Figure 1). The median expression of *MGMT* mRNA was 50.98 (range: 0.0–404.07). We then categorized samples in two groups: low (less or equal than 50.98) or high (higher than 50.98) *MGMT* mRNA expression. Accordingly, 30 cases were allocated to the low expression group and 31 to the high expression group (Table 1).

**Table 1:** Association between *MGMT* methylation and mRNA levels.

Methylation	mRNA(Rs, p)
**MS-MLPA A**	−0.518, p<0.001
**MS-MLPA B**	−0.409, p = 0.001
**MS-MLPA av**	−0.531, p<0.001
**qMSP1**	−0.244, p = n.s.s.
**qMSP2**	−0.428, p = 0.001

### *MGMT* Promoter Methylation Analysis

*MGMT* promoter methylation was examined using two different methods: MS-MLPA and qMSP. Methylation ratios obtained with probe MS-MLPA A were generally higher than those obtained with probe MS-MLPA B (p<0.001, Wilcoxon test). When methylation ratios were compared with mRNA expression levels, statistically significant inverse correlations were observed for both probes, although results derived from the MS-MLPA average ratio of both probes (MS-MLPAav) disclosed the best coefficient (Figure 1 and Table 1). Using the previously defined cut-off (0.25), the overall frequency of *MGMT* promoter methylation determined by MS-MLPA was 21.4%1 (Table 2). Importantly, an inverse association between *MGMT* methylation status and expression was also depicted (p<0.001, Chi-Square test). No statistically significant associations with clinical and pathological parameters were detected.

**Table 2:** *MGMT* promoter methylation frequencies.

Assay	Status	Frequency n (%)
**qMSP1**	Methylated	28 (45.9%)
	Unmethylated	30 (49.2%)
	n.a.	3 (4.9%)
**qMSP2**	Methylated	23 (37.7%)
	Unmethylated	35 (57.4%)
	n.a.	3 (4.9%)
**MS-MLPA**	Methylated	13 (21.4%)
	Unmethylated	45 (73.7%)
	n.a.	3 (4.9%)
**mRNA**	Low levels	30 (49.1%)
	High levels	31 (50.9%)

*MGMT* promoter methylation was also examined by qMSP. No significant differences in methylation levels were found between the two different regions assessed (Wilcoxon test). However, only methylation levels determined using the qMSP2 assay revealed a significant inverse correlation with *MGMT* mRNA expression (p = 0.001; r = −0.428) (Table 1). When methylation results were categorized, the frequency of *MGMT* promoter methylation was 45.9% for qMSP1 and 37.7% for qMSP2 (Table 2). Methylated *MGMT* promoter determined by qMSP2, but not by qMSP1, was significantly associated with low *MGMT* mRNA expression (p = 0.008, Fisher’s Exact Test). Interestingly, *MGMT* methylation levels detected by qMSP2 were statistically associated with higher Karnofsky performance status (KPS) (p = 0.032, Mann-Whitney test).

### *MGMT* Copy Number Alterations, Expression and Methylation Analysis

**Figure 2:**
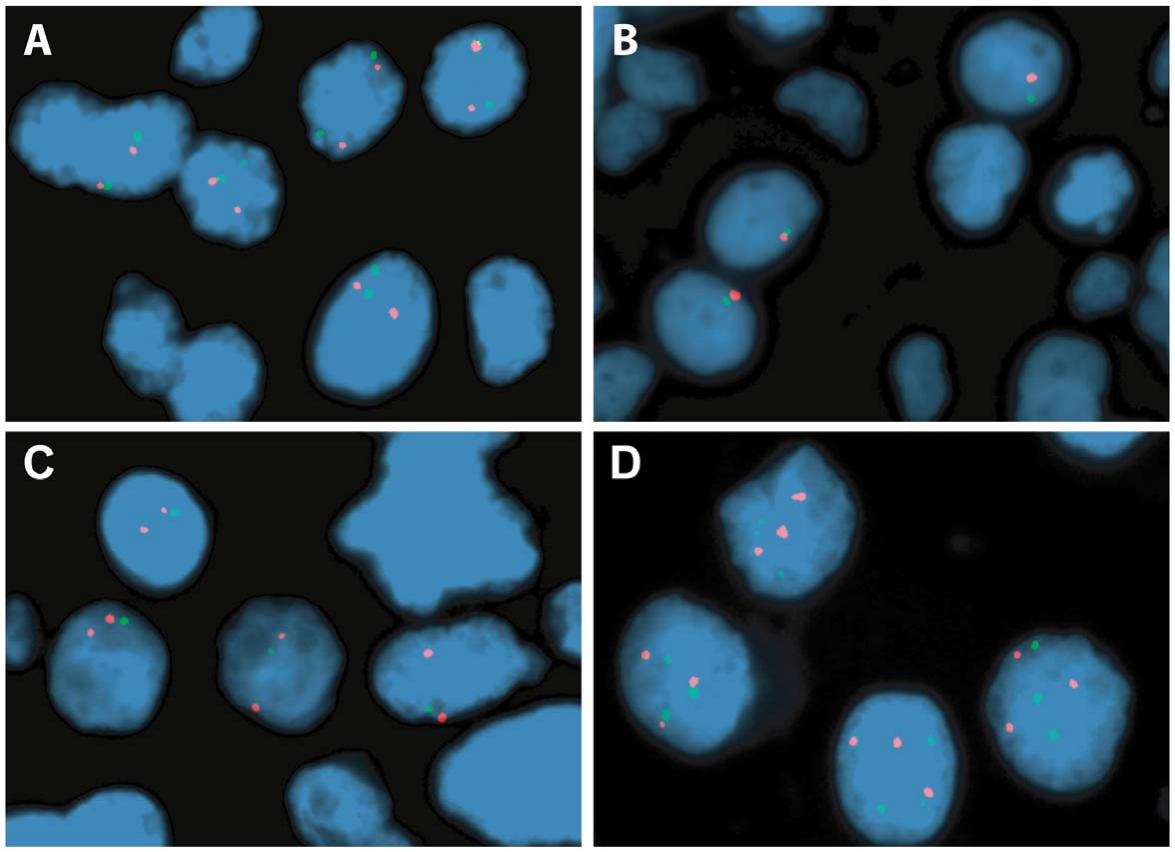
Representative FISH images from high grade gliomas (chromosome 10 centromere probe in red; *MGMT* probe in green). (A) Two copies of chromosome 10 centromere and two copies of *MGMT* (normal). (B) One copy of chromosome 10 centromere and one copy of *MGMT* (monosomy). (C) Two copies of chromosome 10 centromere and one copy of *MGMT* (*MGMT* deletion). (D) Three copies of chromosome 10 centromere and *MGMT* (polysomy).

**Figure 3:**
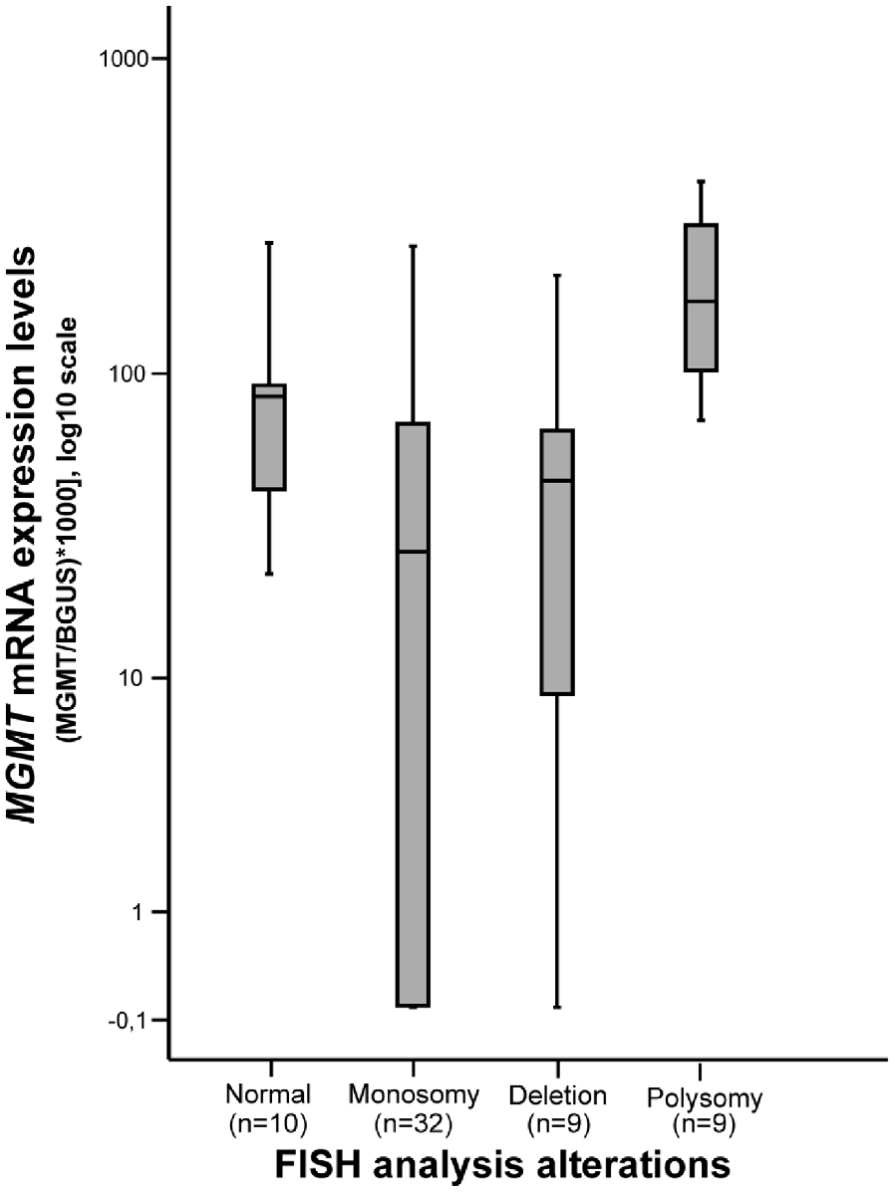
Distribution of *MGMT* mRNA expression levels (log10 transformed) according to the copy number category determined by FISH analysis. Transcript levels of cases with polysomy significantly differed from those with monosomy, deletion or normal (p<0.001, p = 0.002 and p = 0.006, respectively; Mann-Whitney Test).

**Figure 4:**
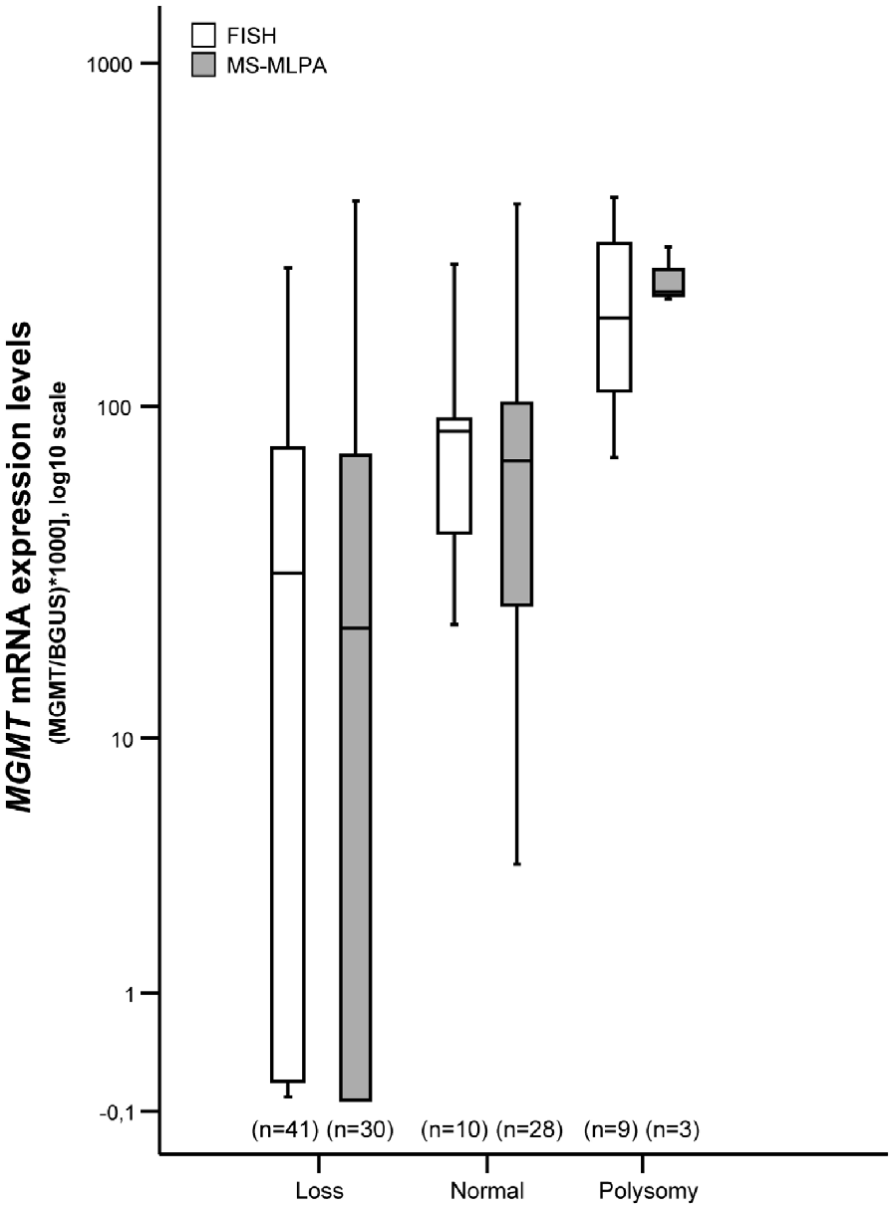
Distribution of *MGMT* mRNA expression levels (log10 transformed) according to the *MGMT* or chromosome 10 copy number category, assessed by FISH or MS-MLPA analysis. Cases with *MGMT* polysomy exhibited significantly higher mRNA levels than cases with *MGMT* loss or normal, examined by FISH (p = 0.006 and p<0.0001, respectively; Mann-Whitney U test) or MS-MLPA (p = 0.013 and p = 0.014, respectively; Mann-Whitney U test).

All samples except one were analyzed for *MGMT* copy number using the FISH probe designed for this study. Tumor cell populations with chromosome 10 monosomy (1∶1) or *MGMT* locus deletion (1∶2) were detected in 32 (53.3%) and 9 (15%) patients, respectively. Chromosome 10 polysomy (3∶3 to 6∶6 signals) was observed in 9 (15%) cases, whereas no alterations were found in 10 (16.7%) samples (Figure 2). Overall, loss of 10q26.1 was identified in 68.3% of all cases. When FISH analysis results were compared with *MGMT* transcript levels, cases with chromosome 10 polysomy displayed the highest mRNA levels, whilst monosomy was associated with lower or absent *MGMT* transcript (p = 0.001, Mann-Whitney test) (Figure 3). Intriguingly, no statistically significant difference in expression levels were apparent among cases with chromosome 10 monosomy, *MGMT* deletion or no copy number alterations, even when the former two groups were coupled as “*MGMT* loss” (Figure 4). No statistically significant associations were apparent when FISH analysis results were compared with clinical and pathological data.

We also evaluated *MGMT* copy number alterations using MS-MLPA. *MGMT* allelic loss was found in 30 (49.2%) cases, 28 (45.9%) cases displayed no *MGMT* copy number alteration, and *MGMT* gain was observed in 3 (4.9%) cases (Figure 4). When these results were compared with *MGMT* mRNA levels, a statistically significant difference among those 3 groups was apparent (p = 0.005, Kruskal-Wallis test) (Figure 4). Comparing FISH and MS-MLPA results, the former technique was able to detect a larger number of alterations. Indeed, in 17 (27.9%) cases loss or gain of *MGMT* was not identified by MS-MLPA, although it was detectable by FISH.

Finally, we assessed the relation between copy number alterations detected by FISH and the promoter methylation analysis. Both qMSP1 and qMSP2 methylation levels were not significantly associated with copy number alterations. However, MS-MLPAav methylation levels significantly correlated with copy number alterations (p = 0.006, Kruskal-Wallis test). Tumors with polysomy displayed the lowest *MGMT* promoter methylation levels, which significantly differed from those of tumors with monosomy, MGMT deletion or with no alterations (normal) (p<0.001, p = 0.009, p = 0.009, respectively; Mann-Whitney test). The same trend was observed when cases with monosomy or MGMT deletion were grouped together (p<0.001; Mann-Whitney test).

### Impact of Epigenetic and Genetic Alterations in *MGMT* Expression

**Figure 5:**
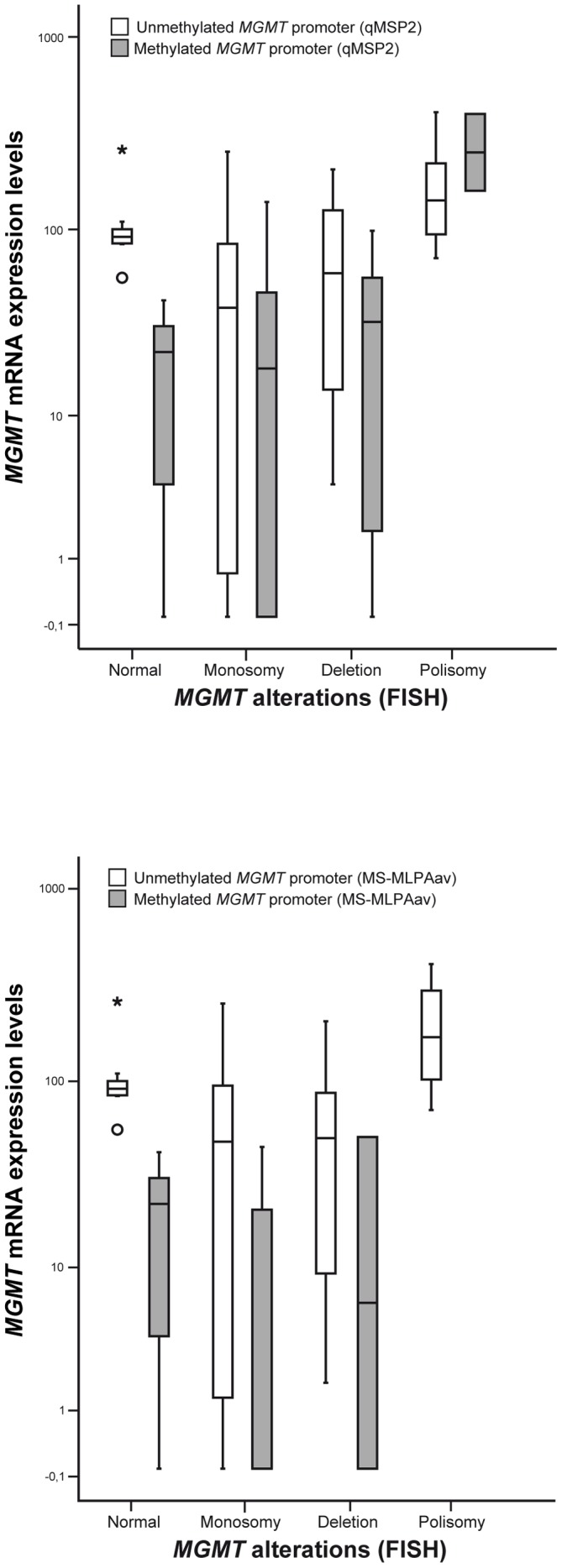
Distribution of *MGMT* mRNA expression levels (log10 transformed) according to *MGMT* promoter methylation status evaluated by (A) qMSP2 or (B) MS-MLPA, in each *MGMT/*chromosome 10 copy number category assessed by FISH analysis. This analysis was not performed for qMSP1 because the methylation results did not correlate with *MGMT* expression (* stands for an outlier value).

After assessing the individual effect of promoter methylation and copy number alterations at the *MGMT* locus in mRNA expression, we sought to determine the combined impact of epigenetic and genetic alterations (Table 3 and Figure 5). As expected, cases methylated at the *MGMT* promoter generally exhibited lower levels of transcript and the same trend was apparent for cases displaying *MGMT* loss (monosomy or deletion). However, in cases with polysomy, the impact of *MGMT* promoter methylation in mRNA expression seems to be null. Conversely, the lowest levels of transcript are observed in cases with combined *MGMT* monosomy or deletion and promoter methylation.

**Table 3:** *MGMT* mRNA levels derived from the interplay between genetic and epigenetic alterations Methylation quantified using A)MS-MLPA or B) qMSP2.

Methylation Status (MS-MLPA)			
**FISH**	**Normal**	Non Methylated	7 (12.1%)
		Methylated	3 (5.2%)
	**Deletion**	Non Methylated	6 (10.3%)
		Methylated	3 (5.2%)
	**Monosomy**	Non Methylated	24 (41.4%)
		Methylated	6 (10.3%)
	**Polysomy**	Non Methylated	9 (15.5%)
		Methylated	0 (0%)
**Methylation Status (qMSP2)**			
**FISH**	**Normal**	Non Methylated	7 (12.1%)
		Methylated	3 (5.2%)
	**Deletion**	Non Methylated	2 (3.4%)
		Methylated	6 (10.3%)
	**Monosomy**	Non Methylated	19 (32.8%)
		Methylated	12 (20.7%)
	**Polysomy**	Non Methylated	7 (12.1%)
		Methylated	2 (3.4%)

### Impact of *MGMT* Alterations in Patient Survival and Prognosis

In this series, median overall survival was 9 months (ranging from 1 to 108 months). Age <58 years, KPS ≥60, and treatment with chemotherapy were associated with prolonged overall survival (p<0.001, p<0.001 and p = 0.034, respectively, Log Rank analysis). We were unable to find any correlation between survival and *MGMT* promoter methylation (determined by MS-MLPA or qMSP), copy number alterations (FISH) or mRNA levels.

In univariate proportional hazards model analysis (Table 4) age ≥58 years (p = 0.001, HR = 2.781; CI: 1.546–5.003), KPS ≥60 (p = 0.001, HR = 0.360; CI: 0.201–0.643), and treatment with chemotherapy (p = 0.043, HR = 2.024; CI: 1.024–4.002) were correlated with patient survival. The multivariate Cox proportional hazard model was computed using selected factors. This analysis revealed that age, KPS and *MGMT* promoter methylation assessed by qMSP1 were independent predictors of progression free survival (PFS) (Table 4). The adjusted hazard ratios of PFS were consistent with the unadjusted hazard ratios.

**Table 4:** Favorable prognostic factors (uni- and multivariate analysis).

Univariate	HR (95%CI)	P
**Age (<58/≥58)**	2.781 (1.546;5.003)	0.001
**Chemotherapy**	2.024 (1.024; 4.002)	0.043
**KPS (cut-off 60)**	0.360 (0.201;0.643)	0.001
**Multivariate (1)**	**HR (95%CI)**	**P**
**Age (<58/≥58)**	3.763 (1.902;7.444)	<0.001
**KPS (<60/≥60)**	0.241 (0.120;0.487)	<0.001
**qMSP1 ** ***MGMT*** ** methylation**	0.470 (0.252;0,875)	0.017

## Discussion

GBM, the most common primary brain tumor in adults, is highly malignant and mostly resistant to currently available therapy [20]. The median survival time is only 12 to 15 months, despite the use of aggressive treatment comprising surgery, postoperative radiotherapy, and adjuvant alkylating agent-based chemotherapy. AA is much less frequent than GBM but it is also an aggressive neoplasm associated with considerable morbidity and mortality. Therapeutic response to alkylating agents, such as temozolomide, is variable, but tumors with *MGMT* promoter methylation have been found to have an increased response rate [31]. Because this epigenetic alteration is associated with gene silencing, we hypothesized that alternative genetic mechanisms, including chromosome 10 monosomy and *MGMT* locus deletion, might lead to *MGMT* downregulation and also predict improved response to chemotherapy.

There is ongoing discussion about the best method to assess *MGMT* dowregulation in gliomas for its use as predictive biomarker of response to alkylating agent therapy, including promoter methylation analysis, levels of mRNA or protein expression, or enzyme activity [4]. Promoter methylation analysis has been the most widely used, although its assessment by qMSP or MS-MLPA (the most commonly performed methods) yields some discordant results [4], [32]. A major cause lies on the CpG sites interrogated by each assay, which are assumed to represent the methylation status of the whole CpG island at the *MGMT* promoter region. Thus, we attempted to identify the CpG sites that could serve as surrogate markers and determine which method (qMSP or MS-MLPA) is more suitable for that purpose. Using the correlation analysis with *MGMT* mRNA expression, the MS-MLPA assay yielded the best results, especially when the average values of the two probes were considered. In addition, MS-MLPA allowed for copy number estimation of *MGMT* alleles. However, the CpG sites assessed by MS-MLPA did not overlap those of qMSP analysis (Figure S1). Furthermore, MS-MLPA requires a larger quantity of high-quality template DNA, which can be difficult to obtain, especially from formalin-fixed, paraffin-embedded tissue samples, in stereotactic biopsy samples and in particular in cases of GBM where necrosis is often extensive. Moreover, as discussed below, the accuracy of copy number estimation by MS-MLPA is limited. Furthermore, recent studies have emphasized that pyrosequencing is more sensitive than qMSP for detection of low levels of methylation [33]–[35]. Nonetheless, this is a more expensive method, not accessible in most routine labs, and it also requires fresh-frozen tissue which is not routinely available. This may account for the fact that qMSP is considered the standard method to test *MGMT* methylation in a clinical setting although the definition of the more clinically significant cut-off value remains a challenge [4,31,32,34, although the definition of an appropriate cut-off value remains a challenge {Preusser, 2009 #31].

The correlation analysis between *MGMT* promoter methylation and mRNA expression levels also indicates that this epigenetic alteration is not the sole responsible for the functional status of the gene. Surprisingly, although the region assessed by qMSP1 was the most frequently methylated in our analysis, it did not significantly correlate with mRNA expression levels. These results are in line with those of Malley *et al*, although pyrosequencing was used instead of qMSP [18]. Indeed, those authors have found that methylation at CpGs located between −250 bp to +240 bp relative to *MGMT* TSS are significantly associated with lower mRNA expression [18]. Remarkably, this region encompasses the location of both MS-MLPA and qMSP2 probes, which were inversely associated with *MGMT* mRNA expression. On the other hand, the genomic region assessed by the qMSP1 assay is characterized by high nucleosome occupancy whereas the qMSP2 region corresponds to a gap between two nucleosomes [18] and is thus considered a major regulatory region of transcription [6], [18]. These data strongly suggest that qMSP2 might provide a more clinically relevant assessment of MGMT promoter methylation.

There is, however, conflicting data concerning the impact of *MGMT* promoter methylation in GBM patient’s outcome [4]. Recently, Everhard and co-workers reported that tumors with unmethylated MGMT promoter showed low levels of mRNA, whereas some tumors with MGMT methylation were found to express high levels of transcript [6]. These data raised the hypothesis that *MGMT* transcriptional activity is not controlled by promoter methylation only in a substantial number of GBM [6]. Our data concerning copy number alterations affecting the *MGMT* locus clearly show that genetic alterations also have an impact in *MGMT* mRNA levels, especially chromosome 10 polysomy (detected in 15% of our cases), which is associated with the highest levels of transcript. This finding is more apparent when FISH analysis is performed for assessment of copy number alterations, compared to MS-MLPA. Indeed, in a sizeable proportion of cases MS-MLPA did not detect alterations and classified cases as “normal”. This is readily apparent in Figure 4, where the distribution of *MGMT* mRNA levels in the “normal” group is much wider for MS-MLPA results than those of FISH analysis. It should also be emphasized that the frequency of total or partial loss of 10q in high-grade gliomas in our study, determined by FISH, is within the range of previous reports [20], [36]. Thus, FISH analysis might be considered the standard method for assessment of copy number alterations at the *MGMT* locus in formalin-fixed paraffin-embedded tissue samples [21]. Since all samples were subjected to macrodissection, it is not likely that the differences observed in FISH vs. MS-MLPA analyses are due to tumor cell sampling, but instead to differences in the sensitivity of the techniques.

An intriguing finding was the lack of significant differences in *MGMT* mRNA expression levels among cases with chromosome 10 monosomy, MGMT locus deletion or normal copy number. This may be explained by the effect of *MGMT* promoter methylation, which is depicted in Figure 5. Because the *MGMT* mRNA levels are similar in the aforementioned 3 groups of tumors, it is reasonable to assume that *MGMT* promoter methylation affects both alleles in “normal” cases. Thus, both genetic and epigenetic alterations contribute to *MGMT* silencing in high-grade gliomas. Conversely, when *MGMT* promoter is unmethylated, cases with monosomy or *MGMT* deletion display lower mRNA expression levels than the cases without copy number alterations, suggesting *MGMT* haplo-insufficiency. On the other hand, chromosome 10 polysomy seems to overcome the silencing effect of *MGMT* promoter methylation, probably because this epigenetic alteration does not affect all alleles. This is consistent with the absence of significant differences in MGMT mRNA expression levels between methylated and unmethylated cases with concomitant chromosome 10 polysomy. This finding, which has not been previously reported, might account, at least partially, for the lack of response to alkylating agents of some patients with GBM carrying *MGMT* promoter methylation [4].

Unexpectedly, cases displaying chromosome 10 polysomy had lower *MGMT* promoter methylation levels. This result suggests that promoter methylation does not uniformly affect all *MGMT* alleles in polysomic tumors, having a relatively low impact in transcript levels. On the other hand, we found that aberrant promoter methylation is a more effective gene silencing mechanism in cases with just one or two *MGMT* alleles. These findings emphasize that genetic changes may significantly confound the effect of promoter methylation, as previously acknowledged by other researchers [37], and that *MGMT* downregulation is the result of a combination of altered genetic and epigenetic mechanisms in high-grade gliomas.

A major limitation of our study is the relatively small size of the group of patients analyzed, which is likely to account for the absence of correlations between *MGMT* promoter methylation, copy number alterations or mRNA levels and overall survival. Furthermore, three out of the five anaplastic astrocytoma samples did not display methylation, irrespective of the method used for assessment, and this result might also have a negative impact in the statistical significance of the survival analysis. This has prevented us from adequately determine whether combined epigenetic and genetic analysis might be more predictive of response to chemotherapy than *MGMT* promoter methylation analysis alone. It has been emphasized that MGMT protein expression is the best predictor of response to Temozolomide [29]. However, this conclusion was drawn from *in vitro* studies and, thus far, no clinically satisfactory assay for assessing MGMT protein in tumor tissues, including immunohistochemistry, has been reported [32]. Thus, it is likely that a combined assay assessing both genetic and epigenetic alterations affecting the *MGMT* locus may serve as a surrogate marker for MGMT protein expression alterations and provide a more useful clinical tool for GBM patient management.

In conclusion, we show here that both epigenetic (promoter methylation) and genetic (monosomy, locus deletion) alterations affecting *MGMT* are associated with lower *MGMT* mRNA levels in high-grade gliomas. Our findings further suggest cooperation between genetic and epigenetic events for effective *MGMT* silencing, which might be more predictive of tumor sensitization to alkylating agents, such as Temozolomide. Moreover, the selection of CpG sites for assessment of promoter methylation is probably more relevant than the method used to assess it. Thus, the chosen methodology should be tailored for each lab depending on the tissue available and the expertise with techniques designed to assess CpG methylation. Finally, evaluation of copy number alterations involving the *MGMT* locus should be incorporated into future trials designed to assess the prognostic and predictive value of MGMT downregulation in patients with high-grade gliomas.

## Supporting Information

Figure S1 **Overview of probes location used for the** ***MGMT*** **promoter methylation assays.**
(PDF)

Table S1 **Primers and probes used for qMSP analysis.**
(DOC)

Table S2 **Clinical and pathological data of the patients.**
(DOCX)
