# Supplementary material for: Altered Expression of MGMT in High-Grade Gliomas Results from the Combined Effect of Epigenetic and Genetic Aberrations
Source: PLoS One. 2013 Mar 11;8(3):e58206. doi: 10.1371/journal.pone.0058206 (PMC3594314; doi:10.1371/journal.pone.0058206)
Supplement: Figure S1 — Overview of probes location used for the MGMT promoter methylation assays. (PDF) [file pone.0058206.s001.pdf]

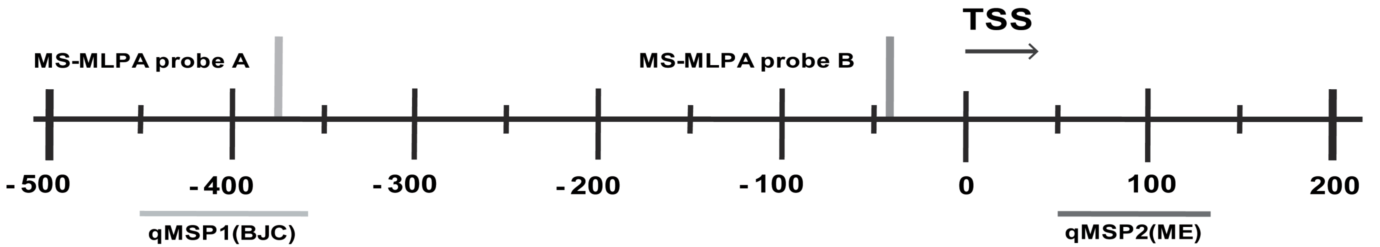

Abbreviations: TSS - transcription start site; qMSP - quantitative methylation-specific PCR; MS-MLPA - Methylation-specific MLPA
