# Supplementary material for: Altered Expression of MGMT in High-Grade Gliomas Results from the Combined Effect of Epigenetic and Genetic Aberrations
Source: PLoS One. 2013 Mar 11;8(3):e58206. doi: 10.1371/journal.pone.0058206 (PMC3594314; doi:10.1371/journal.pone.0058206)
Supplement: Table S1 — Primers and probes used for qMSP analysis. (DOC) [file pone.0058206.s002.doc]

**Supplementary Data**

Table S1. Primers and probes used for qMSP analysis.

| **Name**  **(Genbank)** | **Primer Sequence (5′−3′)** | **bp from TSS** | **Amplicon (bp)** |
| --- | --- | --- | --- |
| ***MGMT* qMSP1**  **(X61657.1)** | Fw: GATTTTTATTAAGCGGGCGTC  Rv: CTTTTCCTATCACAAAAATAATCCG  P: JOE-TCCTAAAAACGCGCGAAAATCGTAAAA-BHQ1 | −476 → −456  −392 → −368  −453 → −428 | 109 |
| ***MGMT* qMSP2**  **(X61657.1)** | Fw: TTTCGACGTTCGTAGGTTTTCGC  Rv: GCACTCTTCCGAAAACGAAACG  P: 6FAM-TGCGTATCGTTTGCG-MGB | 51 → 73  110 → 131  76→ 90 | 81 |
| ***ACTB* qMSP**  **(Y00474)** | Fw: TGGTGATGGAGGAGGTTTAGTAAGT  Rv: AACCAATAAAACCTACTCCTCCCTTAA  P: 6FAM-ACCACCACCCAACACACAATAACAAACACA-TAMRA- | 390 → 414  496→ 522  432→ 461 | 133 |
