# Supplementary material for: Altered Expression of MGMT in High-Grade Gliomas Results from the Combined Effect of Epigenetic and Genetic Aberrations
Source: PLoS One. 2013 Mar 11;8(3):e58206. doi: 10.1371/journal.pone.0058206 (PMC3594314; doi:10.1371/journal.pone.0058206)
Supplement: Table S2 — Clinical and pathological data of the patients. (DOCX) [file pone.0058206.s003.docx]

**Table S2. Clinical and pathological data of the patients**

| **Number of patients** | | **Frequency (n, %)** |
| --- | --- | --- |
| **Age** | <58 | 33 (54.1%) |
|  | ≥58 | 28 (45.9%) |
| **Sex** | male | 32 (52.5%) |
|  | female | 29 (47.5%) |
| **Histology** | GBM | 56 (91.8%) |
|  | AA | 5 (8.2%) |
| **KPS** | <80 | 43 (70.5%) |
|  | ≥80 | 11 (18.0%) |
|  | n.a. | 7 (11.5%) |
| **Radiotherapy** | Performed | 48 (78.7%) |
|  | Not performed | 10 (16.4%) |
|  | n.a. | 3 (4.9%) |
| **Chemotherapy** | Performed | 34 (55.7%) |
|  | Not performed | 14 (30.0%) |
|  | n.a. | 13 (21.3%) |
| **Therapy (RT+ChT)** | Performed | 31 (50.8%) |
|  | Not performed | 28 (45.9%) |
|  | n.a. | 2 (3.3%) |
|  |  |  |

**RT-Radiotherapy; ChT-Chemotherapy**
